# Real-world efficacy of anti-IL-5 treatment in patients with allergic bronchopulmonary aspergillosis

**DOI:** 10.1038/s41598-023-32246-8

**Published:** 2023-04-04

**Authors:** Katsuyoshi Tomomatsu, Hirotaka Yasuba, Takashi Ishiguro, Shiro Imokawa, Johsuke Hara, Seiko Soeda, Norihiro Harada, Naomi Tsurikisawa, Naohiro Oda, Shigeki Katoh, Takanori Numata, Yasuteru Sugino, Mitsuhiro Yamada, Mitsuhiro Kamimura, Takeshi Terashima, Naoki Okada, Jun Tanaka, Tsuyoshi Oguma, Koichiro Asano

**Affiliations:** 1 Division of Pulmonary Medicine, Department of Medicine, Tokai University School of Medicine, 143 Shimokasuya, Isehara, Kanagawa 259-1193 Japan; 2 Department of Airway Medicine, Mitsubishi Kyoto Hospital, Kyoto, Japan; 3 Department of Respiratory Medicine, Saitama Cardiovascular and Respiratory Center, Saitama, Japan; 4 Department of Respiratory Medicine, Iwata City Hospital, Shizuoka, Japan; 5 Department of Respiratory Medicine, Kanazawa University Hospital, Ishikawa, Japan; 6 Department of Allergy and Respiratory Medicine, The Fraternity Memorial Hospital, Tokyo, Japan; 7 Department of Respiratory Medicine, Faculty of Medicine and Graduate School of Medicine, Juntendo University, Tokyo, Japan; 8 Department of Pulmonology, Yokohama City University Graduate School of Medicine, Kanagawa, Japan; 9 Department of Internal Medicine, Fukuyama City Hospital, Hiroshima, Japan; 10 Department of General Medicine, Kawasaki Medical School, Okayama, Japan; 11 Division of Respiratory Diseases, Department of Internal Medicine, The Jikei University School of Medicine, Tokyo, Japan; 12 Department of Respiratory Medicine, Toyota Memorial Hospital, Aichi, Japan; 13 Department of Respiratory Medicine, Tohoku University Graduate School of Medicine, Miyagi, Japan; 14 Department of Pulmonology, National Hospital Organization Disaster Medical Center, Tokyo, Japan; 15 Department of Respiratory Medicine, Tokyo Dental College Ichikawa General Hospital, Chiba, Japan

**Keywords:** Asthma, Medical research

## Abstract

Despite standard treatment with systemic corticosteroids and/or antifungal triazoles, a substantial proportion of patients with allergic bronchopulmonary aspergillosis (ABPA) experience frequent relapses and require long-term treatment despite unfavorable adverse effects. We investigated the efficacy and safety of anti-interleukin (IL)-5/IL-5 receptor α chain (Rα) monoclonal antibodies (mAbs) in patients with ABPA complicated by asthma. ABPA cases treated with anti-IL-5/IL-5Rα mAbs were collected from 132 medical institutes in 2018 and published case reports in Japan. Clinical outcomes, laboratory and physiological data, and radiographic findings during 32 weeks before and after treatment were retrospectively evaluated. We analyzed 29 cases of ABPA: 20 treated with mepolizumab and nine with benralizumab. Treatment with anti-IL-5/IL-5Rα mAbs reduced the frequency of exacerbations (*p* = 0.03), decreased the dose of oral corticosteroids (*p* < 0.01), and improved pulmonary function (*p* = 0.01). Mucus plugs in the bronchi shrank or diminished in 18 patients (82%). Despite the clinical/radiographical improvement, serum levels of total IgE, the key biomarker for the pharmacological response in ABPA, were unchanged. Anti-IL-5/IL-5Rα mAbs that directly target eosinophils are promising candidates for the treatment of patients with ABPA, especially those with mucus plugs in the bronchi.

Allergic bronchopulmonary aspergillosis (ABPA) is an allergic airway disease caused by *Aspergillus fumigatus* or other *Aspergillus* spp. that colonize the bronchi of patients with asthma or cystic fibrosis^1,2^. It is estimated that 2.5–5.0% of asthmatics worldwide develop ABPA^3,4^. It presents with various clinical symptoms, such as worsening asthmatic symptoms, cough, viscous sputum, and radiographic abnormalities in the chest, including pulmonary infiltrates and mucus plugs in the central bronchi. As the disease progresses, it is complicated by irreversible destruction of the airways, such as central bronchiectasis^5^, and secondary infection with *Pseudomonas aeruginosa* or non-tuberculous *Mycobacterium*^6–10^.

The standard treatment for ABPA is the administration of systemic corticosteroids and/or antifungal triazoles^11^. Medium-to-high doses of systemic corticosteroids can achieve disease remission in most cases; however, a substantial proportion of patients (13.5–45%) relapse and eventually become corticosteroid-dependent^12,13^. Long-term treatment with oral corticosteroids causes problems, especially in patients with comorbidities, such as diabetes mellitus or chronic infection in the lower airways. The use of triazole antifungal agents alone or in combination with systemic corticosteroids is another treatment option for ABPA^14,15^. However, post-treatment relapses are still common for this class of drugs, and long-term treatment may induce the emergence of drug-resistant fungi^16^. Therefore, the development of new treatment strategies with fewer adverse effects is warranted for ABPA. As ABPA is characterized by type 1 hypersensitivity to fungi and peripheral blood eosinophilia, biologics targeting type 2 inflammation, developed for the treatment of severe asthma, are expected to be potential therapeutic candidates for ABPA.

We and other researchers have previously reported the efficacy and safety of omalizumab for ABPA accompanied by asthma^17–20^. However, the effects of omalizumab on radiographic findings, such as mucus plugs in the bronchi, are marginal^17^. Furthermore, the dose of omalizumab is often suboptimal owing to markedly high levels of IgE in the serum, which is characteristic of ABPA^17,20–23^. However, a therapeutic strategy to suppress eosinophilic inflammation by targeting interleukin (IL)-5 or its receptor is not hindered by the magnitude of peripheral blood eosinophilia. Two types of antibodies target the IL-5/eosinophil pathway: anti-IL-5 monoclonal antibodies (mAbs), such as mepolizumab and reslizumab, and anti-IL-5 receptor-alpha chain (IL-5Rα) mAbs, such as benralizumab. Anti-IL-5/IL-5Rα mAbs has been shown to be effective against refractory eosinophilic lung diseases such as eosinophilic granulomatosis with polyangiitis, chronic eosinophilic pneumonia, and eosinophilic bronchiolitis^24,25^. Successful treatment outcomes in patients with ABPA have been demonstrated using these modalities^10,26–32^. Benralizumab exhibits more direct anti-eosinophilic effects via its antibody-dependent cell-mediated cytotoxicity on cells expressing IL-5Rα^33^, and some case reports have suggested better clinical outcomes in patients with asthma and ABPA treated with benralizumab than in those treated with mepolizumab^34–36^. However, most data on the efficacy and safety of anti-IL-5/IL-5Rα mAbs for patients with ABPA are based on case reports, except for one case series from Belgium^26^. Herein, we retrospectively analyzed the clinical outcomes, laboratory and physiological data, and radiographic findings of 29 Japanese patients with ABPA complicated by asthma who were treated with mepolizumab or benralizumab.

## Results

### Patient profiles

Twenty-nine patients with ABPM treated with anti-IL-5/IL-5Rα mAbs, including 22 (76%) women with a median age of 63 years, were registered from 15 clinical centers in Japan. All patients were positive for *A. fumigatus*-specific IgE and satisfied the diagnostic criteria for ABPA. The median score for the criteria was 8 (interquartile range [IQR], 5–10); all but one case had a score of 6 or higher, compatible with definite ABPA. The patient demographics and clinical characteristics are summarized in Table 1. All patients had a history of asthma, and there were no cases of cystic fibrosis. The median ages at the onset of ABPA and asthma were 56 and 36 years, respectively.

**Table 1:** Demographic data of the study subjects.

	All	Mepolizumab	Benralizumab	*p*
n (%)	29 (100)	20 (69)	9 (31)	
Age, years	63 (53–70)	64 (57–72)	61 (48–67)	0.32
Female, n (%)	22 (76)	17 (85)	5 (56)	0.16
Asthma, n (%)	29 (100)	20 (100)	9 (100)	–
Age of asthma onset (years)	36 (10–49)	45 (24–56)	11 (8–36)	0.04
Age of ABPA onset (years)	56 (49–66)	59 (50–66)	52 (46–64)	0.44
*Laboratory data at diagnosis*				
Peripheral blood eosinophil counts (/µL)	1495 (977–2430)	1823 (1031–3346)	1166 (830–1525)	0.006
Serum IgE levels (IU/mL)	1066 (554–3187)	1033 (512–2718)	2984 (554–11,028)	0.15
Specific IgE or immediate skin reaction to *A. fumigatus*, n (%)	29 (100)	20 (100)	9 (100)	–
Precipitin to *A. fumigatus*, n (%)	18 (62)	12 (60)	6 (67)	1.00
Fungal culture, n (%)	12 (41)	9 (45)	3 (33)	0.69
Fungal hyphae in mucus plugs, n (%)	7 (24)	6 (30)	1 (11)	0.38
*Thoracic CT*				
Central bronchiectasis, n (%)	26 (90)	18 (90)	8 (89)	1.00
Mucus plugs, n (%)	25 (86)	19 (95)	6 (67)	0.07
High attenuation mucus, n (%)	15 (52)	12 (60)	3 (33)	0.22
*Laboratory data at anti-IL-5/IL-5Rα mAb treatment*				
Peripheral blood eosinophil counts (/µL)	831 (198–1890)	916 (320–2777)	245 (87–1305)	0.11
Serum IgE levels (IU/mL)	888 (193–1798)	603 (177–1021)	1758 (315–9595)	0.05
*Lung function*				
FEV_1_/FVC (%)	71 (60–78)	71 (62–79)	63 (43–78)	0.36
FEV_1_, % predicted	89 (56–101)	90 (64–109)	60 (47–101)	0.35
*Thoracic CT at anti-IL-5/IL-5Rα mAb treatment*				
Mucus plugs, n (%)	22 (76)	17 (85)	5 (56)	0.15
*Therapy at anti-IL-5/IL-5Rα mAb treatment*				
Oral corticosteroids, n (%)	15 (52)	9 (45)	6 (67)	0.42
Dose of oral corticosteroids, mg prednisolone/day	5 (4–20)	5 (3.3–8.8)	15 (4–30)	0.18
Dose of inhaled corticosteroids, μg budesonide/day	800 (570–1600)	800 (425–1280)	1000 (800–1600)	0.18
Antifungal agents, n (%)	10 (35)	6 (30)	4 (44)	0.67
Omalizumab, n (%)	7 (24)	5 (25)	2 (22)	0.63

Values are represented as the median (interquartile range) or n (%).

Twenty patients (69%) were treated with mepolizumab and nine with benralizumab. The age at asthma onset and peak peripheral eosinophil blood counts were higher in the mepolizumab group than in the benralizumab group; however, there were no apparent differences in other patient characteristics between the two treatment groups. The median duration of anti-IL-5/IL-5Rα mAb treatment was 76 weeks (81 and 55 weeks for mepolizumab and benralizumab, respectively). During the administration of anti-IL-5/IL-5Rα mAbs, 15 patients (52%) were on treatment with systemic corticosteroids and 10 (35%) with antifungal drugs. Seven patients had been previously treated with omalizumab, however, the control status of ABPA had worsened when treatment with anti-IL-5/IL-5Rα mAbs was introduced.

### Clinical outcomes

**Figure 1:**
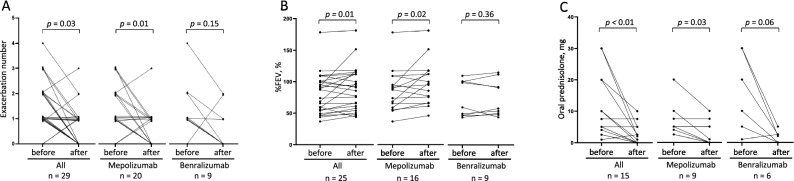
Clinical outcomes of patients. Number of exacerbations within 32 weeks (A, n = 29), percent forced expiratory volume in one second (FEV_1_) of predicted values (B, n = 25), and dose of oral prednisolone (C, n = 15) before and after initiating treatment with mepolizumab or benralizumab in patients with allergic bronchopulmonary aspergillosis complicated by asthma. Treatment with mepolizumab or benralizumab significantly reduced exacerbation rate and oral corticosteroid dose, and improved pulmonary functions.

There was a significant reduction in the exacerbation rate. During the first 32 weeks of anti-IL-5/IL-5Rα mAb treatment, there were 0.62 ± 0.78 exacerbations that required the administration or increase in the dose of systemic corticosteroids, which was 55% less frequent than the rate observed in the 32 weeks before treatment (1.38 ± 1.02, *p* = 0.03, Fig. 1A). Treatment with mepolizumab and benralizumab was similarly effective in decreasing the exacerbation rate (56 vs. 54%, respectively). The median percent forced expiratory volume in one second (FEV_1_) of predicted values increased from 87 to 92% with the anti-IL-5/IL-5Rα mAb treatment (p = 0.014, Fig. 1B). Of the 15 patients treated with oral corticosteroids, the maintenance dose was reduced in 11 patients (73%); the median dose of prednisolone (PSL) was reduced from 5 (IQR, 4–20) mg/day to 2 (IQR, 0–5) mg/day (*p* < 0.01, Fig. 1C). The median dose of PSL was reduced from 5 to 1 mg/day in the mepolizumab treatment group (*p* = 0.03) and from 15 to 2 mg/day in the benralizumab treatment group (*p* = 0.06).

### Biomarkers

**Figure 2:**
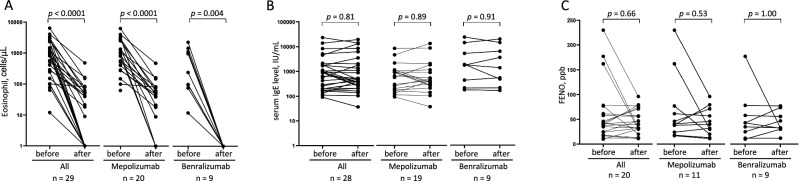
Biomarkers. Peripheral blood eosinophil counts (A, n = 29), serum IgE levels (B, n = 28), and fraction of exhaled nitric oxide (F_E_NO) (C, n = 20) before and after initiating treatment with mepolizumab or benralizumab in patients with allergic bronchopulmonary aspergillosis complicated by asthma. Peripheral blood eosinophil counts were decreased significantly after treatment with mepolizumab or benralizumab, whereas there was no significant change in the serum IgE levels or F_E_NO.

The median peripheral eosinophil count at the start of mepolizumab treatment was 916 cells/µL, which decreased to 45 and 48 cells/µL at 16 and 32 weeks, respectively (*p* < 0.0001, Fig. 2A). The median peripheral eosinophil count at the start of benralizumab treatment was 245 cells/µL, which decreased to 0 cells/µL at 16 and 32 weeks (*p* < 0.004). Meanwhile, there was no significant change in the total IgE levels in the serum or fraction of exhaled nitric oxide (F_E_NO) during the 32-week treatment period (Fig. 2B and C).

### Radiographic findings

Mucus plugs were observed in the central bronchi on thoracic computed tomography (CT) in 22 patients (76%) treated with biologics: 17 of them were treated with mepolizumab and five with benralizumab. Improvement in mucus plugs after treatment was achieved in 18 (82%) patients, including 13 patients (76%) treated with mepolizumab and all patients (100%) treated with benralizumab. In the patients with an improvement of mucus plugs in the bronchi, there was a significantly larger reduction of peripheral blood eosinophil counts after anti-IL-5/IL-5Rα mAb treatment than in those without radiographical improvement (*p* < 0.001, Table 2), even when only the cases treated with mepolizumab were analyzed.

**Table 2:** Peripheral eosinophil counts before and after anti-IL-5/IL-5Rα mAb treatment and improvement of mucus plugs.

	Mucus plugs		*p*	*p* (ANCOVA)
	Improved	Not improved		
*Mepolizumab/Benralizumab*				
n	18	4		
Pre-treatment eosinophil counts, cells/μL	1218 (527–2612)	623 (114–3313)	0.59	0.001
Post-treatment eosinophil counts, cells/μL	35 (0–61)	233 (67–341)	0.02	
Eosinophil reduction rate, %	98.5 (95.3–100)	82.4 (34.5–89.1)	0.001	
*Mepolizumab*				
n	13	4		
Pre-treatment eosinophil counts, cells/μL	1303 (683–2938)	623 (114–3313)	0.41	0.001
Post-treatment eosinophil counts, cells/μL	48 (21–66)	233 (67–341)	0.08	
Eosinophil reduction rate, %	97.9 (90.7–98.8)	82.4 (34.5–89.1)	0.006	

Values are represented as the median (interquartile range).

*AVCOVA* analysis of covariance.

### Switch treatment from mepolizumab to benralizumab

In this study, seven (35%) of the 20 patients originally treated with mepolizumab were switched at 40 weeks (median) to benralizumab treatment. The switch treatment was mostly performed to extend the intervals of treatment, but not for the failure to control respiratory symptoms due to ABPA and asthma with mepolizumab, except for one patient. However, residual mucus plugs in the bronchi were present in six patients despite mepolizumab treatment. After switching to benralizumab, symptoms further improved in two patients (29%); lung function improved in two patients (29%), and mucus plugs disappeared in four (67%) of six patients. No patient showed worsening ABPA or asthma control after switching to benralizumab.

### Safety

There were no severe adverse events, including episodes of anaphylaxis, during treatment with anti-IL-5/IL-5Rα mAbs. One patient developed a temporary headache but continued to receive mepolizumab without recurrence of side effects.

## Discussion

Mepolizumab and benralizumab were approved for the treatment of severe asthma in Japan in 2016 and 2018, respectively. In the present study, we retrospectively evaluated the efficacy and safety of these anti-IL-5/IL-5Rα mAbs in 29 Japanese patients with asthma-complicated ABPA. Mepolizumab, an anti-IL-5 mAb, and benralizumab, an anti-IL-5Rα mAb, were equally effective in reducing the exacerbation rates and doses of oral corticosteroids and improving lung function. Benralizumab may be more effective than mepolizumab in removing mucus plugs from the airways; residual mucus plugs during mepolizumab treatment disappeared in two-thirds of cases after switching to benralizumab. Among type 2 biomarkers, peripheral blood eosinophil counts decreased with these treatments, whereas serum IgE levels, a well-established biomarker for disease activity in ABPA, remained unchanged despite significant clinical improvements^37,38^. There was one case of a mild headache; however, no other serious adverse events, such as anaphylaxis, were observed.

Our data demonstrated that anti-IL-5/IL-5Rα mAbs not only improved clinical control but also radiographic abnormalities, such as mucus plugs in the bronchi. It is often difficult to evaluate whether biologics improve ABPA-specific pathology or underlying asthmatic condition. In contrast, mucus plugs in the central bronchi, often accompanied by central bronchiectasis and high-attenuation mucus, are specific for ABPA^39,40^. Although mucus plugs are also present in the bronchi of severe asthma, they are located in the distal airways and are not accompanied by bronchiectasis or high-attenuation mucus^41^. In the present study, mucus plugs reduced in size or disappeared after treatment with anti-IL-5/IL-5Rα mAbs in 82% of patients, suggesting that these biologics directly affect the pathophysiology of ABPA. Crosslinking of cysteine thiol groups in mucin partially mediated by eosinophil peroxidase has been demonstrated to be important for the formation of mucus plugs in the asthmatic airways^41^, and possibly in the airways of ABPA. In addition, we previously demonstrated that *A. fumigatus* can induce the release of extracellular traps from eosinophils, which are abundant in the mucus plugs of patients with ABPA^42^. Extracellular traps from activated eosinophils, composed of nuclear chromatin, form denser and more stable aggregates than neutrophil-derived extracellular traps and are also present in the mucus plugs of other eosinophilic diseases, such as chronic rhinosinusitis with nasal polyps and eosinophilic otitis media^43^. These data suggest that IL-5 and eosinophils are indispensable for the formation and maintenance of mucus plugs in patients with ABPA.

In the present study, 20 patients initially received mepolizumab, and nine received benralizumab. The clinical effects of mepolizumab and benralizumab on ABPA with respect to the control of daily symptoms or exacerbations were equivalent. However, we found a substantial difference between these biologics in their effects on mucus plugs. Although not statistically significant, benralizumab treatment resulted in a higher rate of disappearance of mucus plugs than mepolizumab treatment. Furthermore, the patients with radiographical improvement on mucus plugs demonstrated significantly larger reduction rate of peripheral blood eosinophil counts. In addition, a switch therapy from mepolizumab to benralizumab diminished the residual mucus plugs on treatment with mepolizumab in four patients, including the two patients we had previously reported^34^. The potent effect of benralizumab in suppressing mucus plug maintenance in the airways is likely related to its robust ability to deplete eosinophils in peripheral blood and tissues. In refractory eosinophilic asthma, bronchial subepithelial eosinophil counts after one year of treatment with mepolizumab were not statistically different from those in the placebo treatment group^44^. In contrast, subcutaneous benralizumab significantly reduced the airway mucosal eosinophil counts, with no eosinophils observed in the mucosal and submucosal tissues at day 84 in the benralizumab group^45^. These differences in the effects of mepolizumab and benralizumab in reducing the airway eosinophil numbers may have resulted in the difference in their effect on ABPA mucus plugs. Removal of eosinophilic mucus plugs by strict management of airway eosinophils with benralizumab prevents airway destruction and improves the long-term prognosis of patients with ABPA.

Various classes of biologics are effective in the treatment of ABPA. We and other researchers have demonstrated that omalizumab is effective in reducing exacerbations and the dose of oral corticosteroids^17,20^. Therefore, patients with ABPA refractory to standard treatment or those with comorbidities, such as diabetes mellitus and persistent airway infection, can benefit from omalizumab treatment. However, the dose of omalizumab needs to be adjusted based on the serum total IgE levels; therefore, sufficient neutralizing activity cannot be expected in cases with markedly high levels of serum IgE. In our previous study, 40% of patients with ABPA were treated with a suboptimal dose of omalizumab in Japan^17^. Seven patients who had been treated with omalizumab received anti-IL-5/IL-5Rα mAbs in this study, with some additional clinical benefits. Anti-IL-5/IL-5Rα mAbs may be more efficacious against mucus plugs than omalizumab. Radiographic improvement was observed in 50% of patients treated with omalizumab in our previous study^17^, whereas it was observed in 82% of patients treated with mepolizumab/benralizumab in this study. Interestingly, anti-IL-5/IL-5Rα mAb treatment was effective without a decrease in the total IgE levels in the serum, although serum IgE levels have been considered essential biomarkers reflecting the disease activity^37,38^. These findings suggest that both IgE and eosinophils are important therapeutic targets for ABPA, and eosinophil-targeted therapy may act directly on eosinophilic mucus plugs.

There have been case reports of ABPA treated with dupilumab, an anti-IL-4Rα mAb, which showed therapeutic effects on the symptoms and pulmonary function^46–48^. Some patients with ABPA refractory to treatment with omalizumab or mepolizumab responded to dupilumab treatment^48–50^; a clinical trial of dupilumab for ABPA is now underway. However, ABPA is often associated with marked eosinophilia, and there are concerns regarding the safety of dupilumab for ABPA treatment due to the systemic effects of eosinophilia. Post-treatment eosinophilia has been observed in clinical trials of dupilumab for atopic dermatitis^51,52^, and the development of symptomatic eosinophilia, such as eosinophilic pneumonia or eosinophilic granulomatosis with polyangiitis, after treatment with dupilumab has also been observed in patients with asthma or chronic rhinosinusitis with nasal polyps^53–57^. Therefore, appropriate biologics should be identified for each patient with ABPA in future studies.

Our study has some limitations. First, it was a retrospective study, which may have biased the patient selection criteria and overestimated the effects of anti-IL-5/IL-5Rα mAbs on ABPA. So far, no cases have been reported with premature discontinuation of anti-IL-5/IL-5Rα mAbs. Second, because anti-IL-5/IL-5Rα mAbs alone have not yet been approved for ABPA treatment, this study was limited to patients with comorbid asthma. A substantial proportion of the patients with ABPA lack specific predisposing factors, such as asthma or cystic fibrosis, and are termed as having ABPA sans asthma. ABPA sans asthma accounts for 7% of ABPA in India^58^ and 19% in Japan^59^. In addition, although efficacy and safety data were collected up to 32 weeks post-dose, long-term data are unknown, and there is a need for prospective long-term evaluation in the future.

## Conclusion

In this study, anti-IL-5/IL-5Rα mAbs decreased the frequency of exacerbation, reduced the dose of oral corticosteroids, and improved lung function in patients with ABPA complicated by asthma, even in those refractory to omalizumab. There was no clear difference in the efficacy or safety between mepolizumab and benralizumab, although benralizumab may be more potent against mucus plugs. Therefore, anti-IL-5/IL-5Rα mAbs targeting eosinophils may be promising therapeutic candidates for patients with ABPA.

## Methods

### Subjects

In 2018, we sent questionnaires to 132 medical institutes that had participated in a nationwide survey on ABPM in 2013^59^, asking about cases of ABPA with concomitant asthma treated with anti-IL-5/IL-5Rα mAbs. In addition, we asked the researchers in Japan who reported cases of ABPA treated with anti-IL-5/IL-5Rα mAbs to participate in this study^27–29^. Clinical data were retrospectively collected from medical records, additional questionnaires to physicians, and a case review meeting held in January 2019.

ABPA was diagnosed based on the modified Asano criteria^39^: (1) current or previous history of asthma or asthmatic symptoms, (2) peripheral blood eosinophilia (≥ 500 cells/mm^3^), (3) elevated total serum IgE levels (≥ 417 IU/mL), (4) immediate cutaneous hypersensitivity or specific IgE for *A. fumigatus*, (5) presence of precipitins or specific IgG for *A. fumigatus*, (6) growth of *Aspergillus* spp. in cultures of sputum or bronchial lavage fluid, (7) presence of fungal hyphae in bronchial mucus plugs, (8) central bronchiectasis on CT, (9) presence of mucus plugs in the central bronchi on CT/bronchoscopy or history of mucus plug expectoration, and (10) high attenuation mucus in the bronchi on CT. Patients who met six or more criteria were diagnosed with definite ABPA, and those who met five criteria were diagnosed as probable cases.

### Ethics declarations

This study was approved by the Institutional Review Board for clinical research of the Tokai University Hospital (#18R-290 and #22R-161) and carried out according to the principles embodied in the Declaration of Helsinki of 1965, as revised in Brazil in 2013. The need for informed patient consent was waived by the Institutional Review Board in view of the anonymity of the data and the retrospective observational nature of the study.

### Treatment effects and safety of anti-IL-5/IL-5Rα mAbs

Mepolizumab or benralizumab was administered subcutaneously at an approved dose for severe asthma. In seven cases in which anti-IL-5 and anti-IL-5Rα mAbs were sequentially administered to the same patient, the therapeutic effects were analyzed for the antibody administered first, whereas adverse effects were evaluated for each antibody. The effects of switching treatment from mepolizumab to benralizumab were analyzed as an additional analysis.

Clinical effects were evaluated with (1) exacerbation rates during 32 weeks prior to and after the initiation of treatment, and (2) dose of oral corticosteroids at baseline and 16 weeks after treatment. Exacerbation was defined as an event that required the administration of or an increase in the dose of systemic corticosteroids.

Functional evaluation was performed by comparing the FEV_1_ measured within 32 weeks before and after treatment with anti-IL-5/IL-5Rα mAb levels. Radiographic assessment of mucus plugs in the bronchi was performed using thoracic CT before and 16–52 weeks after treatment.

### Statistical analysis

Numerical data are presented as the mean and standard deviation or median and IQR, and categorical data are presented as numbers and percentages. Categorical variables were compared using Fisher’s exact test, and continuous variables were compared using the Mann–Whitney *U* test or Wilcoxon signed-rank test and analysis of covariance. Statistical analyses were performed using GraphPad Prism (version 5.0; GraphPad Software, La Jolla, CA, USA) and IBM SPSS Statistics (version 26; IBM Corp., Armonk, NY, USA). Statistical significance was set at p < 0.05.

## Data Availability

The dataset used in this study is available from the corresponding author upon request.
